# Stroke after SARS-CoV-2 vaccination: Comment

**DOI:** 10.1016/j.clinsp.2023.100246

**Published:** 2023-07-05

**Authors:** Amnuay Kleebayoon, Rujittika Mungmunpuntipantip, Viroj Wiwanitkit

**Affiliations:** a Private Academic Consultant, Samraong, Cambodia; b Private Academic Consultant, Bangkok, Thailand; c Chandigarh University, Punjab, India; d Joesph Ayobabalola University, Ikeji-Arakeji, Nigeria

Dear Editor, we would like to share ideas on the publication “Stroke seven hours after SARS-CoV-2 vaccination”.^1^ Fiorini et al. noted that an acute, ischemic stroke can occur shortly after a SARS-CoV-2 vaccination. Fiorini et al. mentioned that there are more arguments for than against a causal relationship between vaccination and stroke. Fiorini et al. noted that future anti-SARS-CoV-2 vaccines must become safer for everyone who is vaccinated.^1^ We both think that protecting COVID-19 is vital and that more research is required to solve the underlying clinical problem. Immunization and the beginning of clinical diseases may be linked. Immunization and the beginning of clinical diseases may be linked. It can be difficult to determine the specific patho-immuno-pharmacological link due to ignorance. If a clinical condition is suspected to be related to COVID-19 immunization, a few critical aspects must be considered. The first is the possibility of co-morbidity. If dengue sickness and vaccine are delivered together, for example, a clinical state may not be adequately detected.^2^ It is also critical to look for any previous cases of asymptomatic COVID-19.^3^ A previous COVID-19 pandemic is likely to have influenced the effectiveness and outcomes of the vaccine. The COVID-19 infection may have an impact on clinical outcomes. Without the right laboratory examinations, it may be difficult to completely rule out the consequences of previous asymptomatic illnesses. Genetics is an important supporting factor.^4^ The immune system's reaction to various genetic components may influence how it tackles unpleasant side effects after vaccinations. Understanding how the underlying genetic component affects vaccine efficacy in clinical settings would be really beneficial. Regarding the pathomechanism of the stroke, it is clearly reported that the COVID-19 vaccine might cause an increase in antibody levels, further cause hyperviscosity, and then result in a thrombotic complication.^5^ Last, if the stroke is due to the vaccine, there must be strong evidence to show the increase in blood viscosity after vaccination. The problem might be a pre-vaccination problem. A good example is a recent report on a case of a stroke while waiting for COVID-19 immunization a few minutes before.^6^ This problem must be resolved if more study is to be undertaken. If enough data is collected, researchers may be able to learn more.

## Conflicts of interest

The authors declare no conflicts of interest.
